# Antidepressive-Like Effect of *Aegle marmelos* Leaf Extract in Chronic Unpredictable Mild Stress-Induced Depression-Like Behaviour in Rats

**DOI:** 10.1155/2022/6479953

**Published:** 2022-12-24

**Authors:** Ashwani Sharma, Talever Singh, Devender Pathak, Tarun Virmani, Girish Kumar, Abdulsalam Alhalmi

**Affiliations:** ^1^Rajiv Academy for Pharmacy, PO Chhatikra, Mathura, Uttar Pradesh 281003, India; ^2^School of Pharmaceutical Sciences, MVN University, Palwal, Haryana 121105, India; ^3^Department of Pharmaceutical Science, College of Pharmacy, Aden University, Aden, Yemen

## Abstract

**Background:**

Depression is a psychiatric disorder leading to anhedonia and lack of interest and motivation. Depressive symptoms are triggered by stressful life events, and patients with major depression are at significantly increased risk of attempting suicide. The crucial concern in depression treatment with antidepressant medications is that few weeks are required to show the therapeutic effect along with moderate side effects. The use of herbal medications is a new strategy for the treatment of depression which is often based on medicinal plants.*Aegle marmelos* (L.) Corr. (family: Rutaceae) is reported to have several actions on the central nervous system producing beneficial effects in anxiety, Alzheimer's disease, Parkinson's disease, epilepsy, and convulsion. Thus, the current investigation designed to assess the antidepressant activity of the standardized hydroethanolic extract of *Aegle marmelos* (EAM) leaves in male rats exposed to the chronic unpredictable mild stress (CUMS) paradigm.

**Methods:**

Rats were divided in 5 groups. The control group was not subjected to experimental CUMS paradigm, while 4 other groups were subjected to CUMS paradigm to induce depression-like behaviour from day 1 to day 28. Following the CUMS paradigm, 4 groups were divided as CUMS disease control, CUMS+EAM (150 mg/kg, p.o.), CUMS+EAM (300 mg/kg, p.o.), and CUMS+imipramine (15 mg/kg, p.o.), and treatment was given for seven consecutive days to the respective groups (day 29 to day 35). Behavioural parameters such as open field test, forced swim test, sucrose feeding test, and tail suspension test on day 1, day 28, and day 35 were measured, and biochemical parameters such as plasma corticosterone level, serotonergic system (5-HT, 5-HIAA, and 5-HT/5-HIAA), mitochondrial function, and proinflammatory mediators (TNF-*α*, IL-1*β*, and IL-6) were estimated in hippocampus (HIP) and prefrontal cortex (PFC) regions of the brain on day 35, after the behavioural observations. On the other hand, phytochemical profile of *Aegle marmelos* was done.

**Results:**

On day 35, EAM (300 mg/kg) significantly reduced the immobility time during the tail suspension test from 208.66 ± 4.72 s to 108.83 ± 4.81 s and forced swim test from 200.16 ± 4.12 s to 148.5 ± 4.58 s. It also enhanced the behavioural parameters in the open field test such as ambulation from 26.5 ± 2.14 to 56.5 ± 1.80, rearing from 8.33 ± 0.71 to 19 ± 0.57, time spent in centre from 9.16 ± 0.9 to 17.16 ± 0.79 s, total distance travelled from 2.36 ± 0.12 to 4.68 ± 0.10 m, and anhedonia in the sucrose feeding test from 109.33 ± 1.08 to 135.83 ± 3.91 mL. The stimulation of the HPA axis resulting elevated corticosterone level caused by CUMS was reduced by EAM (300 mg/kg) from 80.12 ± 2.020 to 48.25 ± 2.407 *μ*g/dL. Furthermore, EAM (300 mg/kg) increase CUMS-induced changes in serotonin (5-HT) level in HIP and PFC from 3.132 ± 0.09586 to 4.518 ± 0.1812 and 4.308 ± 0.1593 to 5.262 ± 0.1014 ng/mg protein, respectively. EAM (300 mg/kg) significantly attenuated the CUMS-induced changes in proinflammatory cytokine production and mitochondrial function in HIP and PFC. One group used to determine the acute toxicity as per OECD-23 standard protocol which resulted that 300 mg/kg EAM has no significant acute toxicity. Total phenolic content and total flavonoid content of standardized hydroalcoholic extract of AM was found 95.024 ± 2.431 and 36.820 ± 3.41, respectively, and additional identification tests showed the presence of alkaloids, tannins, saponins, cardiac glycosides, flavonoids, and terpenoids.

**Conclusion:**

On the basis of findings, EAM can be inferred as a potential antidepressant-like effect of this plan in preclinical research.

## 1. Introduction

Depression is a psychiatric problem described as a discouraged state of mind and repugnance for actual work. Burdensome temperament conditions are set apart by lack of care and slow reasoning, as well as psychomotor hindrance signs like a deficiency of interest in regular activities, anhedonia (the powerlessness to feel joy), and a general indifference [1–3]. Depression influences an expected 3.8% of the total population in the world, with 5% of grown-ups and 5.7% of individuals north of 60 years of age. Nearly 280 million people all over the planet experience the ill effects of sadness. Sadness is distinct from typical emotional episodes and transient close-to-home reactions to day-to-day conditions. Misery can be perilous to one's well-being, particularly if it is steady and has a moderate or extreme force. It can make the person who is impacted endure harshly and perform ineffectively at their specific employment, at school, and in the family. Depression can prompt self-destruction or suicide in the direst stress conditions, and per year, about 700,000 individuals die because of suicidal attempts by depressed ones on [4, 5]. A depressive condition is an incessant, chronic intermittent sickness that adversely affects one's personal life quality and general efficiency. Although many antidepressant classes are currently in operation, there really is a critical need for the development of efficient and secure drugs for the treatment of depression due to clinical limitations and side effects [6]. New methods of treating depression are thus necessary.

Several plant sources, including *Calotropis procera* [7], *Bacopa monniera* [8], St. John's wort extract [9], *Asparagus racemosus* [10], and *Withania somnifera*, among others, have been shown to have antidepressant effects [11]. *Aegle marmelos* Linn, a well-recognised historical ayurvedic plant, belongs to the Rutaceae family and has shown potential for the treatments like asthma, anaemia, fractures, healing of wounds, swollen joints, high blood pressure, jaundice, diarrhoea, healthy mind and brain, typhoid, and troubles during pregnancy [12]. The plant is documented to have a variety of therapeutic qualities. It is also referred to as bael, golden apple, and wood apple. *Aegle marmelos* is a moderate size, subtropical plant that usually grows at 1200-meter altitude region. Traditionally, it is reported that dyspepsia, rheumatism, malabsorption, dysentery, and neurological disorders have all been treated using leaves and fruits of *Aegle marmelos* [13, 14]. Alkaloids, coumarins, amino acids, fatty acids, marmesin, scopoletin, terpenoids, and scoparone are just a few of the many classes of chemicals that have been extracted from the various parts of *A. marmelos*. Alkaloids, flavonoids, carotenoids, polysaccharides, phenolics, and terpenoids are examples of secondary metabolites in plants that help to taste, pigment, and wellness providing qualities [15–17]. Moreover, according to Kothari et al., methanolic extract of *A. marmelos* leaves (in combination) along with imipramine (one group) and fluoxetine (another group) showed antidepressant-like effect in TST in mice, but the study does not include any specific depression-like behaviour inducing model such as CUMS and biochemical parameters were not included. This combination therapy evidenced some antidepressive-like effect of *A. marmelos* [18]. In the current study, CUMS model is used to induce the depression-like behaviour and the behavioural activities in TST, OFT, FST, and sucrose preference test were observed, and biochemical parameters like serotonergic system level, plasma corticosterone level, mitochondrial function, and proinflammatory cytokine expression in HIP and PFC were estimated.

Chronic unexpected mild stress (CUMS) is frequently used as a depression-like behaviour inducing model. Antidepressant drugs have been found to significantly reduce many of the detrimental effects of CUMS in prior studies [19, 20]. CUMS makes rats exhibit depressive-like symptoms, such as a decrease of sucrose desire [21], which is recognised as anhedonia, a crucial depressive symptom [22]. It has long been known that the hyperactivity of the hypothalamic-pituitary-adrenal cortical axis (HPA axis) results in an increase in plasma corticosterone levels in depression [23]. According to Jin et al. [24], some antidepressants low the blood levels of corticosterone that depression causes. This suggests that neuroendocrine adjustment may be one of the medications' potential antidepressant effects. Because of this, the model has face validity (CUMS promotes the behavioural changes observable in depressed individuals), predictive validity (antidepressant medications reverse behavioural changes), and good construct validity (as CUMS decline sensitivity in the brain reward system) [19].

The pathology of depression is thought to include the serotonergic system, according to evidence from the literature [25]. Numerous studies have discovered that different antidepressants restrict serotonin (5-HT) absorption, hence lessening the behavioural abnormalities brought about by CUMS [26, 27]. It has been suggested that sustained HPA axis activation might cause mitochondrial damage. Then, it triggers a monoamine oxidase- (MAO-) induced increase in 5-HT flux, which kills serotonergic neurons in several brain areas, including the hippocampus (HIP) and prefrontal cortex (PFC) [28, 29]. In animal models of depression, the BDNF, which is associated with neuronal survival and neurogenesis, is crucial; it is somewhere related with expression of proinflammatory mediators. CUMS and other stress strategies decrease BDNF concentration in the brain, while long-term antidepressant therapy, especially imipramine, modifies BDNF levels throughout the depressive episode [30–32]. The more proinflammatory mediators shown reduced expression of BDNF in the brain. Additionally, a variety of therapeutic approaches have been shown to provide antidepressant efficacy through the BDNF-mediated activities by reducing the effect of proinflammatory mediators [33–35]. These results show a substantial correlation between mitochondrial, serotonergic, and proinflammatory mediators during depression.

Therefore, in the current investigation, the standardized hydroalcoholic leaf extract of *A. marmelos* was assessed to extract all the water-soluble and alcohol-soluble constituents for its neuroprotective ability in CUMS-exposed depressed rats. Some chemical constituents found in Aegle marmelos leaves are imperatorin and isoimperatorin. Imperatorin is soluble in alcohol and the isoimperatorin is soluble in both water and alcohol. In previous studies, it has been reported that hydroalcoholic extracts of Indian medicinal plants can help more in amelioration from oxidative stress [36–38].

## 2. Materials and Methods

### 2.1. Chemicals and Reagents

Imipramine was purchased from Sigma-Aldrich, and all additional HPLC and analytical grade chemicals/reagents were purchased from Merck Pvt. Ltd. in New Delhi and HiMedia Laboratories Pvt. Ltd., Mumbai, India.

### 2.2. Collection of Plant Leaves and Authentication

Fresh *Aegle marmelos* leaves were taken in the Mathura district of Uttar Pradesh (India). On March 31, 2022, the CSIR-National Institute of Science Communication and Policy Research, New Delhi, received the leaves as a specimen, and authentication number of *A. marmelos* leaves is provided as INIScPR/RHMD/Consult/2022/4054-55. The identification was made through macroscopic examinations of the sample, in-depth examination of the literature, and comparison of the sample with real samples kept in the Raw Material Herbarium and Museum in Delhi (RHMD).

### 2.3. Preparation of *A. marmelos* Leaf Extract

*A. marmelos* fresh leaves were plucked and washed twice with water from the tap. The leaves were drained of any remaining water. The leaves were dried for 10 days in the shade before being crushed in a grinder without the use of any extra solvent. In a Soxhlet extractor, the leaf powder was treated in hydroethanol solvent (60 : 40 ratio) for 24 hours before being dried in desiccators. After the process was finished, a little yield of extracted plant material was left after the solvent was evaporated using a rotary evaporator under reduced pressure.

### 2.4. Analysis of Phytochemicals in *A. marmelos* Leaves

#### 2.4.1. Estimation of the Total Phenolic and Flavonoid Content

The total phenolic and flavonoid content of the *A. marmelos* extract (EAM) was determined.

*(1) Estimation of Total Phenolic Content (TPC)*. With a few minor adjustments, using the [39] assaying method was utilised to quantify the total phenolic content in EAM using the Folin-Ciocalteu reagent. By employing gallic acid as a reference, TPC was assessed using the Folin-Ciocalteu reagent test. 1.5 mL of F-C reagent and 1 mL of plant extract were combined. 1.5 mL of a 7 percent Na₂CO₃ solution was added after 5 minutes. The tubes' final capacity was increased to 10 mL with distilled water, and they were let to remain at room temperature for 90 minutes. Using a UV spectrophotometer (Systronics 2203), the sample's absorbance was measured at 760 nm in comparison to the blank. In terms of GAE/g of dry weight, the results were reported as mean ± SD.

*(2) Estimation of Total Flavonoid Content (TFC)*. TFC was assessed using [40] colorimetric test method. Quercetin serves as a benchmark for the aluminium chloride procedure used to determine it. A volumetric flask was filled with 1 mL of the test material and 4 mL of distilled water. After 5 minutes, 0.3 mL of 10 percent aluminium chloride was added, followed by 0.3 mL of 5 percent sodium nitrite. The reaction mixture received 1 mL of 1 M NaOH after 6 min of room temperature incubation. The final volume had been immediately made up to 10 mL with the distilled water. Using a UV spectrophotometer, the sample's absorbance was determined at 510 nm in comparison to a blank. The entire experiment was run three times to ensure accuracy, and the results were presented as mean ± SD in terms of QE/g of dry mass.

Additionally, according to the previous protocols that were applied to other qualitative phytochemical studies of EAM, for the estimation of alkaloids, tannins, saponins, steroids, cardiac glycosides, terpenoids, phlobatannins, flavonoids, and anthraquinones, the previous techniques were followed [41–43].

#### 2.4.2. Tannins

When 200 mg of leaves were boiled in 10 mL of water (distilled) with required drops of FeCl3 added to the filter, tannins could be seen as a bluish black precipitate.

#### 2.4.3. Alkaloids

In 10 mL of methanol, 200 mg of leaves was heated and filtered. Six drops of Dragendorff reagent and 1% hydrochloric acid have been added right away to check for the presence of alkaloids by looking for a brown to red precipitate.

#### 2.4.4. Frothing Test for Saponins

In 200 mg of sample, 5 mL water (distilled) was added. With distilled water, 1/2 mL of filtrate was diluted up to 5 mL and forcefully shaken for 2 min. The presence of saponins will be indicated if stable foam production is seen.

#### 2.4.5. Keller-Kiliani Test for Cardiac Glycosides

Glacial acetic acid (1 mL) bearing a 2-3 drops of FeCl3 was added to 2 mL of filtrate. The addition of H2SO4 (concentrated) to the aforesaid combination resulted in a green-blue colour, if the cardiac glycosides are present.

#### 2.4.6. Steroids (Liebermann-Burchard Reaction)

In chloroform (10 mL), 200 mg of sample was added. The addition of acetic anhydride at the ratio of 1 : 1 resulted in the production of a bluish green ring, indicating steroids' presence.

#### 2.4.7. Terpenoids (Salkowski Test)

2 mL of CHCl3 and 3 mL strong H2SO4 were carefully added in 200 mg of sample. If the terpenoids are present, a red to brown colouring is seen.

#### 2.4.8. Flavonoids

Diluted ammonia solution (5 mL) was added in aqueous filtrate, immediately followed by adding concentrated H2SO4. Yellow colour is the indication for flavonoids.

#### 2.4.9. Phlobatannins

A few drops of 1 percent hydrochloric acid were added to the boiling tube after 200 mg of plant material had been dissolved in 10 mL of aqueous extract; a crimson precipitate is the indication for the presence of phlobatannins.

#### 2.4.10. Anthraquinones

In 10 percent HCl, 500 mg of dried plant material was boiled for 5 minutes before being filtered and left to cool. A similar amount of CHCl3 and several drops of 10% NH3 were added to a 2 mL filtrate. A rose-pink colour is indication for the presence of anthraquinones.

### 2.5. Acute Toxicity Study

The “Organisation for Economic Co-operation and Development” OECD-423 standard guidelines were used to conduct an acute oral toxicity investigation. Adult male albino Wistar rats (*N* = 3, 180-220 grams) were tested. The rats were administered orally extract of *A. marmelos* leaves in doses of 5, 50, 300, and 2000 mg/kg (in 0.5 percent carboxymethyl cellulose (CMC) in the form of suspension). The animals were monitored for 24 hours and then 14 days for fatalities and the appearance of general toxicological signs and symptoms.

### 2.6. Selection of Dose of Standardized EAM

The EAM was found to be tolerated in experimental rats at escalating doses of 5, 50, 300, and up to 2000 mg/kg in an acute oral toxicity study. Furthermore, after the first 3 hours of EAM treatment, the rats showed no gross behavioural, neurological, or autonomic toxic effects, and there was no fatality after 24 to 72 hours till 14 days. At the concentrations used in this investigation, the absence of toxicity symptoms suggests that hydroethanolic EAM leaves were nontoxic and well tolerated. The EAM were given orally in two doses: 150 mg/kg and 300 mg/kg to the body weight. The reference standard drug was imipramine given at a 15 mg/kg dose. Normal saline was given to the control group.

### 2.7. Experimental Animals and Their Housing

Animal house of Rajiv Academy for Pharmacy, Mathura, India, provided adult albino Wistar rats (male) having weight 180-220 grams, aged 10 weeks for the study. The rats were housed in 5 groups in cages and 6 rats in each group were caged (polyacrylic) lined with husk under regular circumstances (temperature: 25 ± 2°C, relative humidity: 45–55%, and a 12 h light-12 h dark cycle). The animals had full access to both their regular pellet diet (provided by Lipton India, Ltd., Mumbai) and water. The animals were not given food for 16–18 hours before the studies, but they were free to consume as much water as they pleased.

### 2.8. Experimental Design

The rats were divided into five groups and each group has 6 rats. Rats were subjected to depression using the CUMS model except the control group. The behavioural examinations of the rats were conducted on the 1st day (day 1). After that, depression-like behaviours were observed on day 28 in rats after CUMS exposure where rats showed depression-like behaviours. One group remained as normal control, one group remained as CUMS (disease control), two groups received EAM (150 and 300 mg/kg, respectively) [18], and one group received imipramine (15 mg/kg) [7] for seven days after the development of depression. One hour later receiving the final dose of EAM (150 and 300 mg/kg) on day 35, all rats underwent antidepressant activity tests in the OFT, TST, FST, and sucrose feeding test. For further investigation into the antidepressant efficacy of EAM for impaired HPA axis high activity, alteration in serotonergic system, proinflammatory cytokine expressions, and impaired mitochondrial function and integrity in the rats' HIP and PFC, the rats were sacrificed by cervical dislocation and the brain was dissected.

### 2.9. Chronic Unpredictable Mild Stress (CUMS) Protocol

With a few changes [44], the CUMS standard approach [19] was used to establish depression-like behaviour. The every week of CUMS cycle out of 4 weeks included one time of coupled caging for 2 hours, one time of tilted cage at a 45° angle well within time frame of wet caging for 3 hours, one time span of food meal deprivation for 18 hours followed by one time frame of tightly restricted access to food for 1 hour, two times of water deprivation followed by one hour of exposure to an empty water bottle, one time of wet cage with 200 mL water in the 100 grams of sawdust bedding for 21 hours, and one time of continuous light exposure for 36 hours. As a result, the rats were exposed to stressors throughout both their active (dark) and inactive (light) phases. The control rats' living quarters and cages were left alone.

### 2.10. Observation of Behavioural Parameters and Biochemical Estimation

#### 2.10.1. Forced Swim Test (FST)

With a few minor modifications, the test was conducted in accordance with the reference guidelines [45, 46]. Swim drills were carried out in clear glass cylinders that were water filled having temperature 23–25°C, 46 cm high, 20 cm diameter, and 30 cm deeply filled with water. Two swim assessments were conducted, each between 10:00 and 16:00, with a pretest lasting 15 minutes and a posttest lasting 5 minutes. After both swimming periods, the rats were removed from the cylinders, dried with paper towels, and kept in warmed cages about 15 minutes just before being put back into their original cages. The forepaws' upward-directed motions along the side of the swim cylinder are referred to as climbing behaviour. Swimming activity is defined as movement (mainly horizontal) throughout the swim cylinder's quadrants as well as across the whole swim cylinder. The behaviours selected for monitoring in the modified FST were those that involved immobility, which is defined as the absence of any extra movement other than that required to keep the rat's head above the water. Every five seconds throughout the test session, the sampling procedure counts the occurrences of each behaviour.

#### 2.10.2. Tail Suspension Test (TST)

The rat was hung by its tail to a steady metal rod, which was 50 cm above the surface, with its body facing down. Normally, animals would attempt to climb up the rod to evade the stressful circumstance, but depressed animals would give up and remain immobile. The duration of immobility throughout the five minutes of this test, which indicated depressive-like behaviour, was assessed [47, 48].

#### 2.10.3. Open Field Test (OFT)

OFT is a popular technique for evaluating rat locomotion and emotional reaction [49]. The field was a prefabricated, 60 × 60 cm, open hardwood field with 30-centimeter-high barriers. Each individual rat was placed in the middle of the open area, and for 5 minutes, its behaviour, including the total travelled distance, immobility period, number of rearing, and number of line crossings with the hind leg, was observed. Grid crossings through the hind legs were measured under the best lighting conditions as a percentage of overall transit distance and rearing as an exploratory behaviour [50, 51].

#### 2.10.4. Sucrose Feeding Test

The method described by [52, 53] for evaluating the desire for sucrose was used. Rats were put into cages one at a time during the experiment. Before use, the rats remained without water for 24 hours. Separate cages were used for the rats, and they had unrestricted access to one bottle containing sucrose solution (1% *w*/*v*) in every cage. For 24 hours, the second bottle was switched out for water. The test of sucrose preference in the early hours was completed. The volume proportion of sucrose solution and water consumed after the test was noted.

#### 2.10.5. Corticosterone Estimation

According to Woodward and Emery's [54] description, the serum corticosterone concentration was measured using an HPLC equipped with an ultraviolet (UV) detection system, with dexamethasone added as a main reference [55]. In essence, 500 *μ*L of plasma containing a particular dosage of dexamethasone was extracted using 5 mL of dichloromethane. The dichloromethane has been diluted in 100 *μ*L of mobile phase after being evaporated till it was entirely dried. 20 microliters of the extract was loaded into the HPLC analyser for measurement. At a flow rate of 1.2 mL/min, a UV detector was used to detect CORT at 250 nm in a mobile phase of methanol/water (70 : 30) (model: LC-20AD, Shimadzu, Japan). To record and analyse the chromatogram, Empower software was utilised.

#### 2.10.6. Brain Dissection

Animal sacrifice was carried out through cervical dislocation. The brain was swiftly removed and immediately cleaned in 0.9% saline solution [56]. HIP and PFC tissues were homogenised in 1 mL of 0.1 M perchloric acid using a homogeniser. The homogenate was put in polypropylene tubes for 15 minutes, and then, 50 *μ*L of 4 M potassium acetate was added to adjust the pH to 4.0, followed by 15 minutes of centrifugation at 4000 *g* [57].

#### 2.10.7. Estimation of Monoamines

Two brain regions, the HIP and PFC, had their levels of neurotransmitters and related metabolites, such as serotonin (5-HT) and 5-hydroxy indole acetic acid (5-HIAA), as well as their ratio, evaluated using HPLC equipped with an ultraviolet (UV) detection system (model: LC-20AD, Shimadzu, Japan) [55, 58].

#### 2.10.8. Estimation of Mitochondrial Function (MTT Assay)

*(1) Isolation Mitochondria from Rat Brain*. Mitochondria had been removed from the HIP and PFC tissues using the standard procedure [59]. To ascertain the amount of mitochondria protein, the standard procedure was adopted [60]. After execution of rats, tissues were removed and placed in an isolation buffer that was ice-cold and included 225 mM mannitol, 5 mM HEPES, 75 mM sucrose, and 1 mg/mL fatty acid free BSA (isotonized with KOH; pH 7.4). The tissue was minced and homogenization done inside a Dounce-type glass homogeniser with isolation buffer (1/10, *w*/*v*) added to 1 mM EDTA as chelator. For 10 minutes, the homogenate was centrifuged at 600 rpm. To recover the fast-sediment mitochondria, the supernatant was collected in a cooled receiver, and the aggregates were resuspended in 10 mL buffer and centrifuged at 600 rpm for 10 minutes. After collecting the supernatants, they were centrifuged at 12,000 rpm, for 8 minutes. After being reconstituted in buffer, the pellets were subsequently centrifuged at 12,000 rpm for 10 minutes. The pellet is composed of layers that vary in firmness and appearance, with a top layer which is whitish and fluffy and comprises lipids, synaptosomes, and myelin. After removing the synaptosomal surface, the brown mitochondrial pellets were collected and reconstituted in buffer but also centrifuged at 12,000 rpm for 10 minutes. In isolation buffer devoid of chelators, the final mitochondria pellets were again resuspended.

*(2) Estimation of MTT Assay (Mitochondrial Function)*. The pellet was suspended in phosphate buffer saline in Eppendorf tube. Add 10 *μ*L MTT in 300 *μ*L mitochondrial suspension and incubate for 30 minutes. The suspension has been centrifuged for five minutes about 12,000 rpm, and the supernatant was carefully thrown away. The sample was dissolved in 200 *μ*L of DMSO (model: LMSF-V320, Spectrophotometer, Labman Scientific Instruments Pvt. Limited). By measuring the formazan produced at 595 nm [61], the 3-(4, 5-dimethylthiazol-2-yl)-2, 5-diphenyltetrazolium bromide (MTT) reduction was utilised to determine the mitochondrial function. The results were presented as mg protein/min/mg formazan produced.

#### 2.10.9. Proinflammatory Cytokine Quantitative Estimation in HIP and PFC

Proteinase blocker (100 mg tissue/mL) was added to phosphate-buffered solution to help homogenise the HIP and PFC tissues. The mixture was then centrifuged at 12,000 rpm for 10 minutes. Tumor necrosis factor (TNF), interleukin-6 (IL-6), and interleukin-1 (IL-1) concentrations throughout the supernatant were measured in accordance with manufacturer recommendations using enzyme-linked immunosorbent assay kit (Abcam Pvt. Ltd., India) spectrophotometrically at 450 nm (model: UV-1800, Shimadzu, Japan). Standardized to total tissue weight, the cytokine concentrations in homogenates were represented as pg/100 mg tissue sample [62].

### 2.11. Data Analysis

Mean and standard deviation first from mean were used to present the data as standard error mean. The statistical analysis was completed using GraphPad Prism 5 software. Analysis of variance (ANOVA) test was used to identify the differences between the groups. Two-way ANOVA followed by the Bonferroni post hoc test was used in FST, TST, OFT, and sucrose preference test, and one-way ANOVA followed by the Newman-Keuls post hoc test was used in estimation of plasma corticosterone, serotonergic system, mitochondrial function, and proinflammatory cytokine expression. Always, ≥5% (*p* < 0.05) level of significance was required to rule out the null hypothesis.

## 3. Results

### 3.1. Analysis of Phytochemicals in *A. marmelos* Leaves

Phytochemical analysis revealed that EAM included saponins, alkaloids, flavonoids, steroids, tannins, and phenols. The TPC and TFC of standardized hydroethanolic extract of AM was determined through spectrophotometric analysis, and results are mentioned in Sections 3.1.1 and 3.1.2, respectively.

#### 3.1.1. Total Phenolic Content

**Figure 1:**
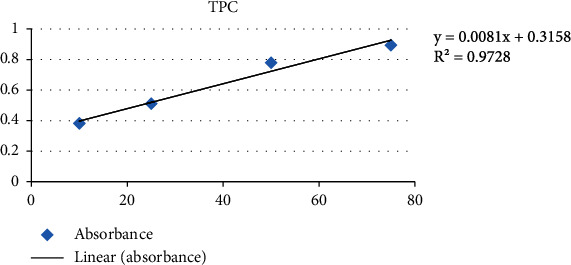
Gallic acid standard curve.

Using a UV spectrophotometer, the sample's absorbance was measured at 760 nm in comparison to a blank, and the standard gallic curve is shown in Figure 1.

The results, which are listed in Table 1, were reported as mean ± SD in terms of GAE/g of dry weight.

#### 3.1.2. Total Flavonoid Content

**Figure 2:**
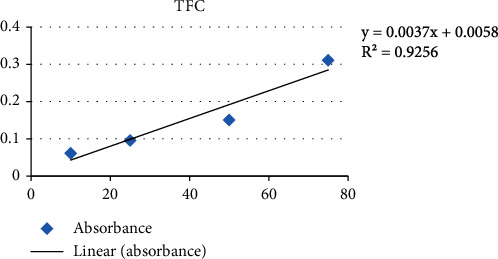
Standard curve of quercetin.

Using a UV spectrophotometer, the sample's absorbance was measured at 510 nm in comparison to a blank, and standard curve of quercetin is shown in Figure 2.

The entire experiment was run three times for accuracy; the results, which are represented in mean ± SD in terms of QE/g of dry weight, are listed in Table 2.

#### 3.1.3. Phytochemical Profiling

In all the types and accessions, hydroethanolic extract indicated the existence of alkaloids, tannins, saponins, cardiac glycosides, flavonoids, and terpenoids, whereas phlobatannins and anthocyanins were lacking as mentioned in Table 3.

### 3.2. Effect of EAM (150 and 300 mg/kg) in CUMS-Induced Rats

#### 3.2.1. Effect of EAM (150 and 300 mg/kg) on CUMS-Induced Changed Behaviours in FST

**Figure 3:**
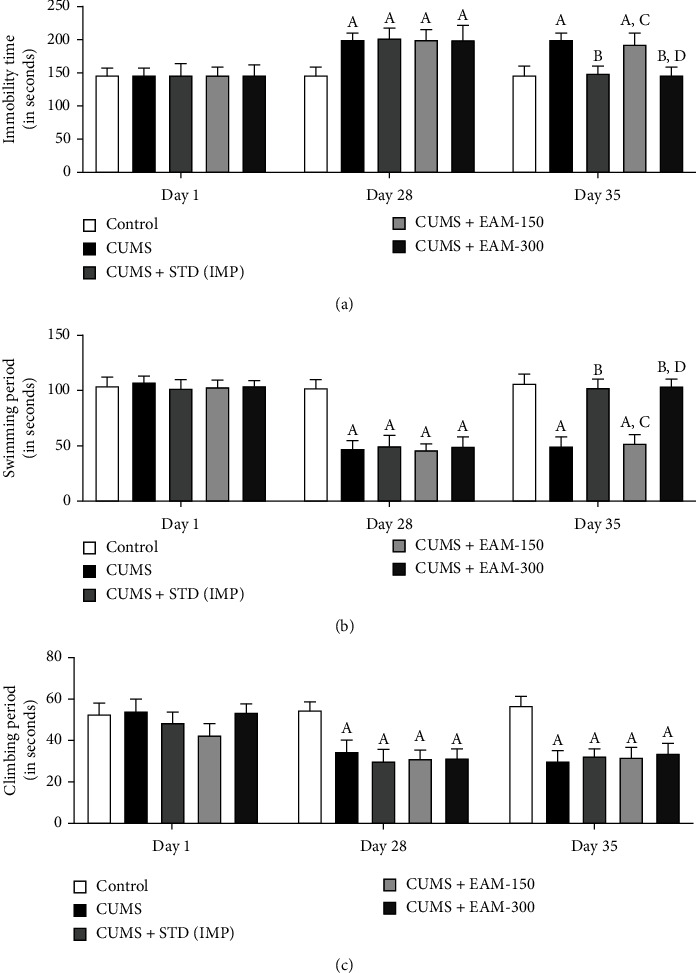
The effect of EAM (150 and 300 mg/kg) against CUMS-induced alterations during FST paradigm in (a) immobility, (b) swimming, and (c) climbing periods at different time points. All the data values are mean ± SEM (*n* = 6). ^A^*p* < 0.001 vs. control, ^B^*p* < 0.001 vs. CUMS, ^C^*p* < 0.001 vs. IMP (15 mg/kg), ^D^*p* < 0.001 vs. EAM 150 mg/kg, and ^E^*p* < 0.001 vs. EAM 300 mg/kg (two-way ANOVA followed by Bonferroni post hoc test).

During FST on day 1 of the experimental procedure, post hoc analysis indicated no considerable variations in climbing, swimming, and immobility time across groups, while all CUMS-exposed rat groups were found to have decreased climbing and swimming period while increased the immobility period on day 28 as compared to the control group. On day 35, EAM (300 mg/kg) and IMP substantially attenuated the increase in immobility brought on by CUMS and decreased swimming and climbing period as compared to the control group. The impact of EAM (150 and 300 mg/kg) against CUMS-induced alterations in the immobility, swimming, and climbing durations of rats in FST is shown in Figures 3(a)–3(c), respectively. Statistic evaluation results showed the considerable variations in climbing, swimming, and immobility periods in the course of FST between the groups ([*F* (4, 75) = 20.68, *p* < 0.001], [*F* (4, 75) = 96.49, *p* < 0.001], and [*F* (4, 75) = 43.99, *p* < 0.001], respectively) and time ([*F* (2, 75) = 79.15, *p* < 0.001], [*F* (2, 75) = 328.6, *p* < 0.001], and [*F* (2, 75) = 82.95, *p* < 0.001], respectively). Furthermore, during the FST, there was a considerable interaction across groups and time during the immobility [*F* (8, 75) = 11.73, *p* < 0.001], swimming [*F* (8, 75) = 50.15, *p* < 0.001], and climbing [*F* (8, 75) = 8.482, *p* < 0.001] periods.

#### 3.2.2. Effect of EAM (150 and 300 mg/kg) against CUMS-Induced Altered Immobility in TST

**Figure 4:**
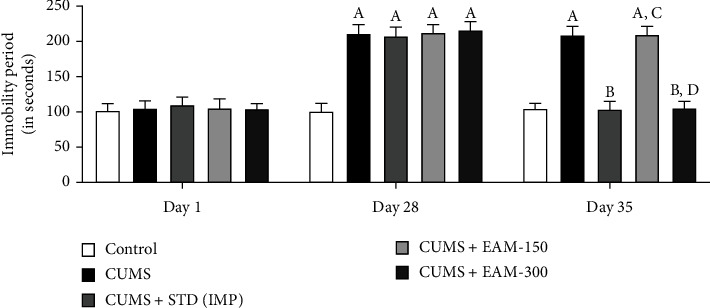
The effect of EAM (150 and 300 mg/kg) against CUMS-induced changes during the TST in immobility duration at different time points. All the values are mean ± SEM (*n* = 6). ^A^*p* < 0.001 vs. control, ^B^*p* < 0.001 vs. CUMS, ^C^*p* < 0.001 vs. IMP (15 mg/kg), ^D^*p* < 0.001 vs. EAM 150 mg/kg, and ^E^*p* < 0.001 vs. EAM 300 mg/kg (two-way ANOVA followed by Bonferroni post hoc test).

In the course of TST on day 1, a post hoc test presented no remarkable changes in immobility time among the groups, while all CUMS-exposed rat groups found to have increased the immobility period on day 28 as compared to the control group. On day 35, EAM (300 mg/kg) and IMP substantially attenuated the increase in immobility brought on by CUMS in reference to the control group.  Figure 4 shows the EAM effect (150 and 300 mg/kg) against CUMS-induced alterations in the immobility duration in the TST protocol. The immobility period during TST differed significantly across groups [*F* (4, 75) = 56.99, *p* < 0.001] and time [*F* (2, 75) = 213.5, *p* < 0.001], according to statistical analysis. Furthermore, during the TST, significant group interactions occurred in immobility duration [*F* (8, 75) = 31.84, *p* < 0.001].

#### 3.2.3. Effect of EAM (150 and 300 mg/kg) on CUMS-Induced Altered Behaviours in OFT

**Figure 5:**
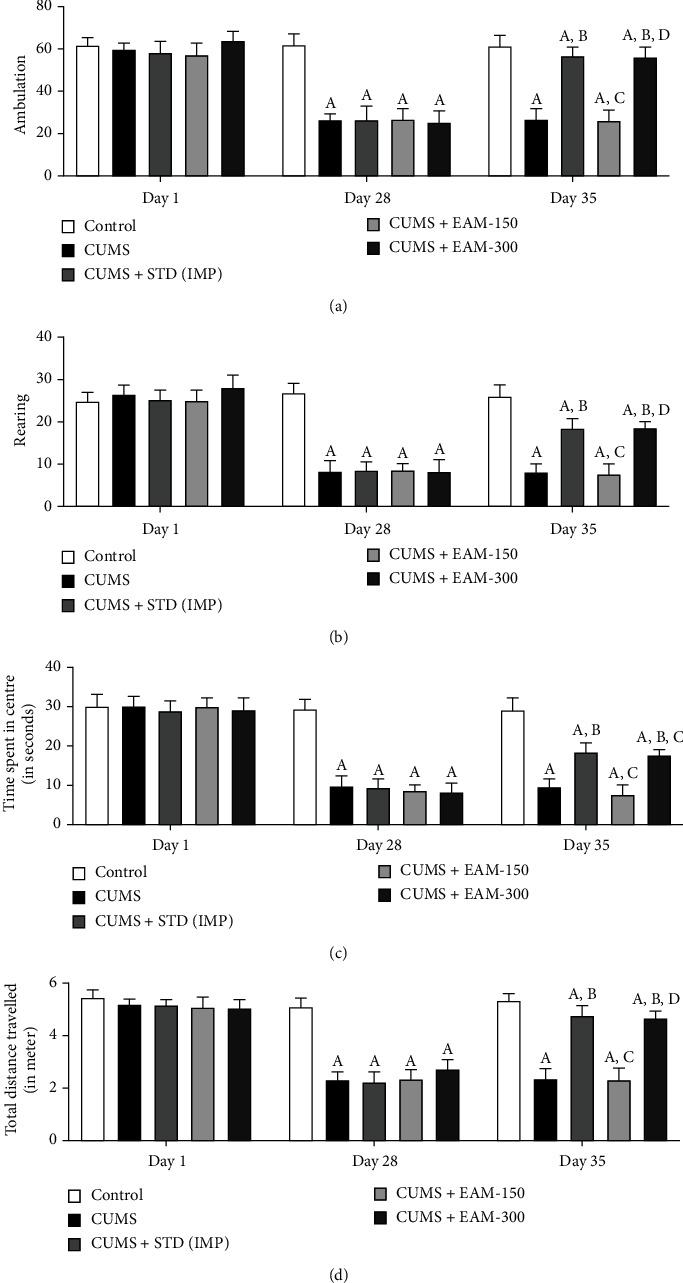
The effect of EAM (150 and 300 mg/kg) on CUMS-induced changes during the OFT in (a) ambulation, (b) rearing, (c) time spent in the centre, and (d) total distance travelled at different time points. All the values are mean ± SEM (*n* = 6). ^A^*p* < 0.001 vs. control, ^B^*p* < 0.001 vs. CUMS, ^C^*p* < 0.001 vs. IMP (15 mg/kg), ^D^*p* < 0.001 vs. EAM 150 mg/kg, and ^E^*p* < 0.001 vs. EAM 300 mg/kg (two-way ANOVA followed by Bonferroni post hoc test).

During OFT on day 1 of the experimental procedure, post hoc analysis indicated no significant changes in all behavioural characteristics across groups. On day 28, CUMS generated a substantial drop in all behavioural measures during the OFT paradigm as compared to the control group. EAM (300 mg/kg) and IMP substantially exacerbated the CUMS reduction every behavioural activity in the course of OFT, on day 35. The impact of EAM (150 and 300 mg/kg) against CUMS induced alterations in ambulation, rearing, time spent in the centre, and total distance travelled in the OFT protocol as depicted in Figures 5(a)–5(d), respectively. Statistic evaluation disclosed significant variations in rearing, time spent in the centre, ambulation, and total distance travelled during OFT between groups ([*F* (4, 75) = 92.76, *p* < 0.001], [*F* (4, 75) = 85.00, *p* < 0.001], [*F* (4, 75) = 106.6, *p* < 0.001], and [*F* (4, 75) = 143.5, *p* < 0.001]), as well as time ([*F* (2, 75) = 258.9, *p* < 0.001], [*F* (2, 75) = 304.2, *p* < 0.001], [*F* (2, 75) = 415.9, *p* < 0.001], and [*F* (2, 75) = 434.1, *p* < 0.001]), respectively. Moreover, during OFT, significant group interactions occurred in time spent ambulating [*F* (8, 75) = 37.97, *p* < 0.001], rearing [*F* (8, 75) = 35.64, *p* < 0.001], time spent in the centre [*F* (8, 75) = 31.86, *p* < 0.001], and total distance walked [*F* (8, 75) = 52.25, *p* < 0.001].

#### 3.2.4. Effect of EAM (150 and 300 mg/kg) on CUMS-Induced Altered Anhedonia Behaviour

**Figure 6:**
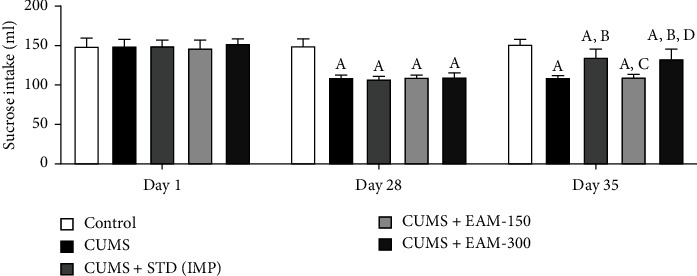
The effect of EAM (150 and 300 mg/kg) against CUMS-occurred changes during the intake of sucrose by the animals at different time points. All the data values are mean ± SEM (*n* = 6). ^A^*p* < 0.001 vs. control, ^B^*p* < 0.001 vs. CUMS, ^C^*p* < 0.001 vs. IMP (15 mg/kg), ^D^*p* < 0.001 vs. EAM 150 mg/kg, and ^E^*p* < 0.001 vs. EAM 300 mg/kg (two-way ANOVA followed by Bonferroni post hoc test).

On day 1 of the experimental methodology, a post hoc test revealed no notable changes in the sucrose intake across groups, while on D-28, CUMS induced a considerable reduction in sucrose intake, which lasted until day 35. EAM (300 mg/kg) and standard drug IMP exacerbated the CUMS-occurred reduction in sucrose intake on D-35. The impact of EAM (150 and 300 mg/kg) against CUMS-occurred changes in terms of sucrose intake is depicted in Figure 6. There were notable variations in the sucrose intake across groups [*F* (4, 75) = 61.56, *p* < 0.001] and time [*F* (2, 75) = 195.6, *p* < 0.001] according to statistical analysis. Furthermore, in terms of sucrose consumption, significant interactions between group and time were observed [*F* (8, 75) = 21.01, *p* < 0.001].

#### 3.2.5. Effect of EAM (150 and 300 mg/kg) against CUMS-Induced Changes in Concentration of Plasma Corticosterone

**Figure 7:**
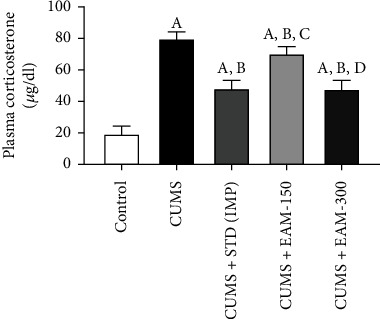
The EAM (150 and 300 mg/kg) effect on CUMS-occurred changes in plasma corticosterone concentration. All the values are mean ± SEM (*n* = 6). ^A^*p* < 0.001 vs. control, ^B^*p* < 0.001 vs. CUMS, ^B^*p* < 0.001 CUMS+300 vs. CUMS, ^B^*p* < 0.05 CUMS+150 vs. CUMS, ^C^*p* < 0.001 vs. CUMS+STD, ^D^*p* < 0.001 vs. EAM 150 mg/kg, and ^E^*p* < 0.001 vs. EAM 300 mg/kg (one-way ANOVA followed by Newman-Keuls post hoc test).

On day 35 of the trial schedule, a post hoc test revealed that CUMS induced a substantial elevation in plasma corticosterone concentrations. The CUMS protocol rise in plasma corticosterone in rats was considerably reduced by EAM (300 mg/kg) and IMP. The impact of EAM (150 and 300 mg/kg) on CUMS-occurred changes in plasma corticosterone levels in the rats is shown in Figure 7. Statistical analysis indicated significant variations in plasma corticosterone levels across groups [*F* (4, 25) = 95.68, *p* < 0.001].

#### 3.2.6. Effect of EAM (150 and 300 mg/kg) on CUMS-Induced Changes Altered Serotonergic Activity in HIP and PFC

**Figure 8:**
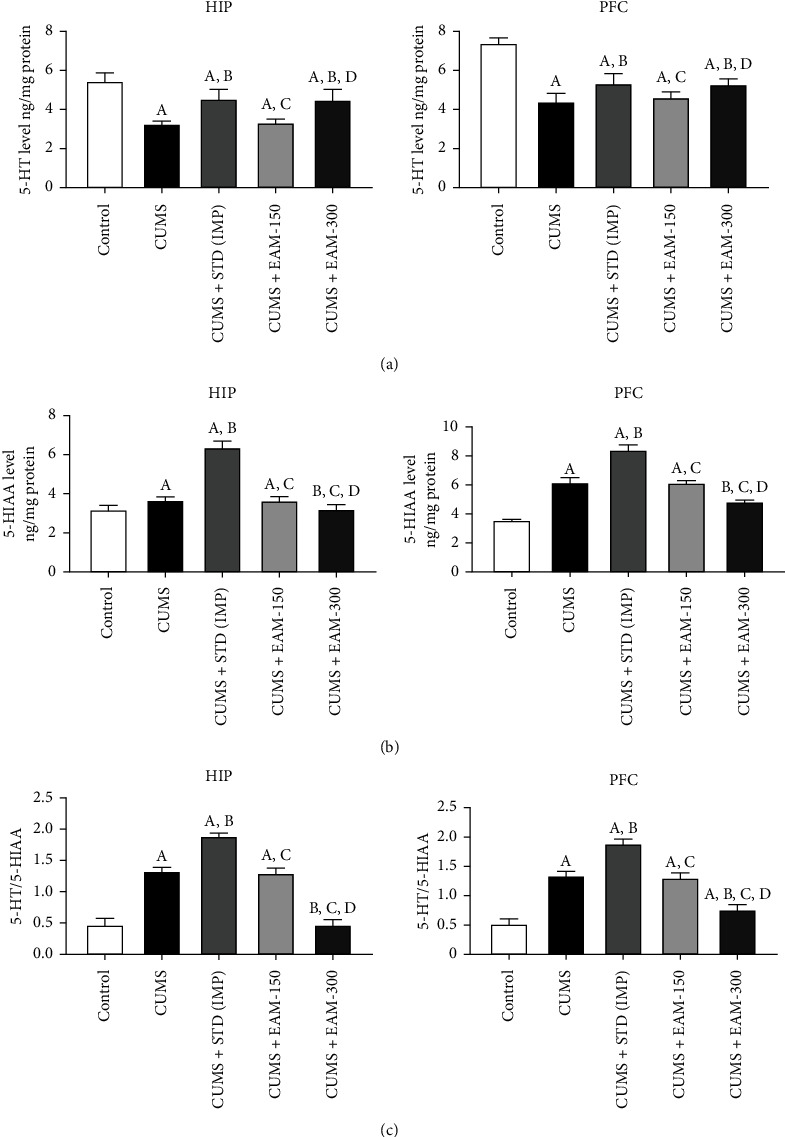
The EAM (150 and 300 mg/kg) effect on CUMS-derived changes in the level of (a) 5-HT and (b) 5-HIAA levels and the ratio of (c) 5-HIAA/5-HT in HIP and PFC. All the data values are mean ± SEM (*n* = 6). ^A^*p* < 0.001 vs. control, ^B^*p* < 0.001 vs. CUMS, ^C^*p* < 0.001 vs. IMP (15 mg/kg), ^D^*p* < 0.001 vs. EAM 150 mg/kg, and ^E^*p* < 0.001 vs. EAM 300 mg/kg (one-way ANOVA followed by Newman-Keuls post hoc test).

The EAM (150 and 300 mg/kg) influence of CUMS-derived changes in 5-HT (Figure 8(a)) and 5-HIAA (Figure 8(b)) levels, as well as the ratio of 5-HIAA/5-HT (Figure 8(c)), is shown. The levels of 5-HT and 5-HIAA, as well as their ratio 5-HIAA/5-HT in HIP ([*F* (4, 25) = 46.57, *p* < 0.001], [*F* (4, 25) = 422.2, *p* < 0.001], and [*F* (4, 25) = 262.3, *p* < 0.001], respectively) and PFC ([*F* (4, 25) = 76.54, *p* < 0.001], [*F* (4, 25) = 407.2, *p* < 0.001], and [*F* (4, 25) = 294.5, *p* < 0.001], respectively). According to a post hoc test, CUMS induced a substantial drop and increases the 5-HT and 5-HIAA in brain areas (HIP and PFC), respectively, when compared to the control group. Furthermore, as compared to the control group, CUMS induced a considerable rise in the ratio of 5-HIAA/5-HT HIP and PFC. In both the brain areas of the rats, EAM (300 mg/kg) and IMP substantially attenuated the CUMS-derived drop in 5-HT levels. In all brain areas, EAM (300 mg/kg) significantly reduced the CUMS-induced rise in 5-HIAA levels. IMP, on the other hand, significantly exacerbated the CUMS-induced rise in 5-HIAA levels in both rat brain areas. EAM (300 mg/kg) also reduced the CUMS-induced rise in the ratio of 5-HIAA/5-HT in all brain regions, while standard drug IMP substantially exacerbated the CUMS-derived rise in the level of 5-HIAA/5-HT in HIP and PFC.

#### 3.2.7. Effect of EAM (150 and 300 mg/kg) on CUMS-Induced Altered Mitochondrial Function MTT Assay in HIP and PFC

**Figure 9:**
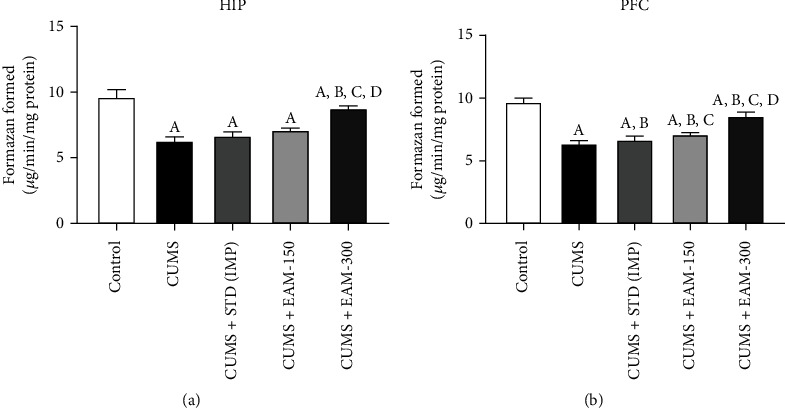
The impact of EAM (150 and 300 mg/kg) on CUMS-derived alterations in (a) mitochondrial function and (b) integrity in HIP and PFC. All values are mean ± SEM (*n* = 6). ^A^*p* < 0.05 vs. control, ^B^*p* < 0.001 CUMS+EAM 300 vs. CUMS, ^B^*p* < 0.05 CUMS+STD vs. CUMS, ^C^*p* < 0.001 vs. CUMS+STD, ^C^*p* < 0.05 CUMS+STD vs. CUMS+EAM 150, ^D^*p* < 0.05 vs. EAM 150 mg/kg, and ^E^*p* < 0.05 vs. EAM 300 mg/kg (one-way ANOVA followed by Newman-Keuls test).

The impact of EAM (150 and 300 mg/kg) against CUMS-derived alterations in mitochondrial function and integrity is shown in Figures 9(a) and 9(b), respectively. The results of the statistical analysis showed that HIP and PFC had significantly different levels of mitochondrial function ([*F* (4, 25) = 382.9, *p* < 0.001] and [*F* (4, 25) = 140.8, *p* < 0.01). A post hoc study showed that CUMS substantially decreased mitochondrial function in PFC and HIP, when compared to the control group. The CUMS decline in mitochondrial activity in both part of the animals' brain areas was greatly mitigated by EAM (300 mg/kg).

#### 3.2.8. Effect of EAM (150 and 300 mg/kg) on CUMS Altered Proinflammatory Cytokine Expression in HIP and PFC

Table 4 shows the influence of EAM (150 and 300 mg/kg) against CUMS-derived changes in (A) TNF-*α* expression, (B) IL-1*β* expression, and (C) IL-6 expression in HIP and PFC. Results of the statistical analysis showed that HIP and PFC had significantly different expression of (A) TNF-*α* expression, (B) IL-1*β* expression, and (C) IL-6 expression in HIP ([*F* (4, 25) = 1176, *p* < 0.001], [*F* (4, 25) = 4639, *p* < 0.001]), and [*F* (4, 25) = 187.9, *p* < 0.001], respectively) and in PFC ([*F* (4, 25) = 2873, *p* < 0.001], [*F* (4, 25) = 6988, *p* < 0.001]), and [*F* (4, 25) = 103.0, *p* < 0.001], respectively). When compared to the control group, a post hoc analysis revealed that CUMS significantly increases the TNF-*α* expression, IL-1*β* expression, and IL-6 expression in HIP and PFC. Furthermore, EAM-300 and IMP significantly attenuated the CUMS-induced increase in the expression of TNF-*α*, IL-1*β*, and IL-6 in HIP and PFC.

## 4. Discussion

The current study reveals that in CUMS-challenged rats, EAM (300 mg/kg) showed antidepressant-like actions. HPA axis hyperactivity brought on by CUMS was reduced by EAM (300 mg/kg). Additionally, EAM (300 mg/kg) attenuated the effects of CUMS-induced alterations in the rats' HIP and PFC's serotonergic and proinflammatory cytokine expression, oxidative stress, mitochondrial function, and integrity. These findings could suggest that EAM could be an alternative for treating depression.

A person's conduct, sense of well-being, thinking, and emotions can all be impacted by the neuropsychiatric illness known as depression. Certain types of depression-like behaviour can occur in both people and animals when they are exposed to persistent stress [63, 64]. CUMS in the current study exacerbated the anhedonia behaviour in the rats' sucrose feeding test. CUMS lengthened the immobility duration in TST and FST, while reducing the animals' climbing and swimming propensities. Additionally, CUMS reduced the rats' rearing, ambulation, time in the centre, and overall distance covered in the OFT compared to the prior data [65]. These results imply that these animals display depressive-like behaviours after being exposed to CUMS. In the current study, EAM at a dosage level of 300 mg/kg diminished all CUMS-induced depression-like behaviours in every test scenario [66–68].

It is interesting to note that EAM (300 mg/kg) boosted the CUMS-induced decline in the swimming behaviour in rats during FST. However, there is one limitation of current study that it has no impact on the way animal climbing behaviour during the FST which had been altered by CUMS while previous studies reported that imipramine attenuates the climbing behaviours altered by CUMS. It has been hypothesized that during FST, animals' swimming behaviour is primarily influenced by serotonergic brain activity [69]. As a result, EAM (300 mg/kg) may have an impact on the serotonergic activity in the brains of CUMS-challenged rodents and consequently have antidepressant-like effects in these rats. There is a substantial amount of clinical and experimental data indicating that monoaminergic systems, particularly serotonin, are crucial in the pathophysiology of depression. According to López-Muñoz and Alamo [70], the common mechanism of the majority of antidepressants now used in clinical settings, including imipramine, is the regulation of serotonergic neurotransmission at the synaptic level. Therefore, the levels of 5-HT and its metabolic product (5-HIAA) were assessed in order to provide evidence in favour of the hypothesis that the antidepressant-like effect of EAM (300 mg/kg) is mediated by the rise in serotonergic activity. The HIP and PFC, which are crucially involved in the regulation of depression and are brain areas that are physically and functionally impacted by stress reactions, received specific focus in the current study [25, 71]. In the current investigation, CUMS significantly increased 5-HIAA levels, decreased 5-HT levels, and increased their turnover (5-HIAA/5-HT) in HIP and PFC, which is consistent with past findings [72–74]. It is interesting to note that EAM (300 mg/kg) lowered the amount of 5-HIAA and 5-HT turnover that CUMS-induced rise in both brain areas of the rats. However, the elevation in 5-HIAA and 5-HT turnover levels driven by CUMS was further accelerated by IMP in both rat brain regions. These findings suggest that EAM (300 mg/kg) improves the serotonergic activity in these brain areas, either by blocking the 5-HT transporter or by increasing the release and synthesis of 5-HT and/or by reducing the activity of the MAO enzyme. This must be confirmed with future research experiments.

Different neuron populations and brain-resident immune cells create and release inflammatory mediators during the acute phase of the disease, which aids in the development of the disorder [75]. During various forms of CNS trauma, proinflammatory cytokines including TNF-*α*, IL-1, and interferon play a critical role in illness development, ultimately leading to decreased energy generation, cell death, and brain damage [76]. In this work, it is found that EAM (300 mg/kg) administration reduced IL-6, IL-1*β*, and TNF-*α* expressions. This shows that EAM, a dose of 300 mg/kg, probably influences neurogenesis by reducing the release of proinflammatory cytokines in the HIP and PFC as a part of its antidepressant action.

EAM (300 mg/kg) in the current research prevented the CUMS-induced decline in mitochondrial integrity and activity in both regions of the brain. It is interesting to note that IMP had no effect on the decline in mitochondrial function and integrity brought on by CUMS in either of the two brain areas. IMP had a positive impact on mitochondria in olfactory bulbectomy-induced depressed and genetically depressed rats, in contrast to current results [77]. The impact of IMP might be a determining factor in how depression-like behaviour is brought on in animals. It is widely recognised that an elevated corticosterone level in brain tissues can cause mitochondrial dysfunction, likely through the glucocorticoid receptor. This can have several negative effects, including an increase in the activity of the MAO enzyme and a decrease in the protein BDNF level in particular brain regions. In the current investigation, in both brain areas of the CUMS-challenged rats, IMP increased 5-HT turnover whereas EAM (300 mg/kg) decreased it. This suggests that in such circumstances, EAM may partially modulate mitochondrial function to have positive effects on the serotonergic system.

These findings highlight the fact that EAM (300 mg/kg), either as a single component or in combination, exerts an antidepressant effect. This is likely accomplished via modulating the HPA axis, which in turn affects mitochondria-dependent serotonergic activity and neurogenesis.

CUMS-induced depression-like behaviours are subjected to some excess and deficiencies in some criteria which regulate the normal behaviour. The main concern in the depression is stress, when the rats subjected to stress then in result HPA axis hyperactivation leads to release cortisol also known as stress hormone which increase the plasma corticosterone level. A second mechanism, rapid feedback, is independent of the precise level of corticosteroids in the brain. However, prolonged exposure to cortisol in the brain causes hippocampal cell loss. Increased levels of corticosterone are a hallmark of depression since all of this is caused by hyperactivity of the HPA axis, which is a result of the stress situation. Furthermore, this will lead to reduction in serotonin level, and increase in proinflammatory mediator expression leads to decreased BDNF in the brain which reduces the neurogenesis and reduces the mitochondrial function in HIP and PFC. This will lead to depression-like behaviours which were observed after CUMS given to rats in FST, TST, OFT, and sucrose preference test. EAM significantly attenuated the depressive behaviours in rats and reduces the HPA axis hyperactivity, increase the availability of serotonin, increase the mitochondrial function, and decrease the proinflammatory cytokine expression which were estimated in the current study as compared to control rats. This is how EAM is effective in CUMS-induced depression-like behaviour in rats.

## 5. Conclusion

Through the findings of the above investigation, it can be concluded that, in CUMS-challenged rats, the hydroalcoholic extract of *Aegle marmelos* leaves significantly showed antidepressant-like action by attenuating the depression-like behaviours during FST, TST, OFT, and sucrose preference test. EAM significantly reduced the CUMS-induced hyperactivity of the HPA axis and attenuated the changes in serotonergic systems, mitochondrial function, and the production of proinflammatory cytokines in the rats' HIP and PFC. On the basis of findings, it is concluded that EAM can be inferred as a potential antidepressant-like effect of this plan in preclinical research.

## Figures and Tables

**Table 1:** Result of TPC.

TPC (mg of gallic acid equivalent/g of dry weight)		
Sample	Solvent	TPC (mean ± SD)
Fine dry powder	Hydroethanolic	95.024 ± 2.431

**Table 2:** Result of total flavonoid content.

TFC (mg of quercetin equivalent/g of dry weight)		
Sample	Solvent of extract	TFC (mean ± SD)
Fine dry powder	Hydroethanolic	36.820 ± 3.41

**Table 3:** Phytochemical profiling of *A. marmelos*.

Chemical constituent	Presence (+)/absence (-) of chemical constituents
Alkaloids	+
Cardiac glycosides	+
Saponins	+
Flavonoids	+
Terpenoids	+
Tannins	+
Phlobatannins	—
Anthocyanins	—

**Table 4:** EAM (150 and 300 mg/kg) effect on CUMS altered proinflammatory cytokine expression in HIP and PFC.

Groups	HIP			PFC		
	TNF-*α*	IL-1*β*	IL-6	TNF-*α*	IL-1*β*	IL-6
Control	74.09 ± 10.96	131.3 ± 15.92	39.48 ± 0.5811	89.59 ± 10.33	234.8 ± 23.19	41.48 ± 0.5811
CUMS	1022 ± 8.134^a^	3623 ± 26.30^a^	68.59 ± 0.6381^a^	2013 ± 13.35^a^	4824 ± 36.50^a^	70.43 ± 1.382^a^
CUMS+STD	632.0 ± 10.46^a,b^	1435 ± 26.01^a,b^	53.20 ± 0.9293^a,b^	805.8 ± 14.84^a,b^	751.5 ± 32.50^a,b^	58.20 ± 0.929^a,b^
CUMS+EAM 150	1012 ± 9.594^a,c^	3624 ± 25.61^a,c^	70.02 ± 0.6425^a,c^	2026 ± 21.06^a,c^	4815 ± 27.24^a,c^	68.59 ± 0.6381^a,c^
CUMS+EAM 300	657.1 ± 15.73^a,b,d^	1453 ± 15.69^a,b,d^	55.68 ± 1.465^a,b,d^	828.1 ± 17.18^a,b,d^	794.6 ± 14.14^a,b,d^	60.68 ± 1.704^a,b,d^

All values are mean ± SEM (*n* = 6) and one-way ANOVA followed by Newman-Keuls post hoc test. ^a^*p* < 0.001 vs. control. ^b^*p* < 0.001 vs. CUMS. ^c^*p* < 0.001 vs. CUMS+STD. ^d^*p* < 0.001 vs. CUMS+150.

## Data Availability

The supporting literature, data, and other necessary information used to support the findings of this study are available from the corresponding author upon request.
